# Attitudes towards receiving COVID-19 vaccine and its associated factors among Southwest Ethiopian adults, 2021

**DOI:** 10.1371/journal.pone.0280633

**Published:** 2023-01-23

**Authors:** Mamo Solomon Emire, Bisrat Zeleke Shiferaw

**Affiliations:** Department of Nursing, College of Medicine and Health Sciences, Wolkite University, Wolkite, Ethiopia; The University of the West Indies, TRINIDAD AND TOBAGO

## Abstract

**Introduction:**

Many countries around the world are still affected by the global pandemic of coronavirus disease. The vaccine is the most effective method of controlling Coronavirus Disease 2019 (COVID-19). However, attitudes toward vaccination are heavily affected by different factors besides vaccine availability.

**Objectives:**

This study aimed to determine community attitudes toward the COVID-19 vaccine in Gurage Zone, Ethiopia.

**Methods:**

A community-based cross-sectional study was conducted from November 15^th^ to December 15^th^, 2021. A simple random sampling technique was used to select 364 participants in the study area. An interview-administered structured questionnaire was used to collect the data; the data was entered into Epidata 3.1 version, and then exported to SPSS version 23 for further analysis. Descriptive statistics were used to determine the characteristics of study participants. Binary and multivariable logistic regression analyses with a p-value of less than 0.05 were used as a measure of significance.

**Results:**

In this study, 44.7% of study participants had a favorable attitude toward the COVID-19 vaccine. Perceived potential vaccine harm [AOR: 1.85; 95% CI (1.15–2.96)], Having ever had a chronic disease [AOR: 3.22; 95% CI (2.02–5.14)], community belief on the effectiveness of the vaccine [AOR: 2.02; 95% CI (1.27–3.22)], and average monthly income 3001–5000 ETB [AOR: 0.54; 95% CI (0.30–0.97)], average monthly income 5001–10000 ETB [AOR: 0.48; 95% CI(0.27–0.86)] were statistically significantly towards COVID-19 vaccination.

**Conclusions:**

Overall, less than half of the participants had a favorable attitude toward the COVID-19 vaccine. Perceived potential vaccine harm, having ever had a chronic disease, community belief in the effectiveness of the vaccine, and average monthly income were determinant factors of the community’s attitude toward COVID-19 vaccination. As a result, information conversation with the community’s awareness of the COVID-19 vaccination in reducing vaccine-related suspicion.

## Background

Coronavirus (CoV) is derived from the Latin word ’corona,’ which means "crown" [1]. It causes a variety of respiratory tract infections in humans, ranging from a moderate cold to severe respiratory distress syndrome [2]. Human-to-human transmission occurs by common routes such as direct transfer; frequent means of spread include coughing, sneezing, droplet inhalation, and contact with oral, nasal, and eye mucous membranes [3].

Different studies on COVID-19 vaccine hesitancy have been published in India 36%, Canada 20%, and the United States 25%, showing that the factors responsible for vaccine hesitancy from socioeconomic demographics, occupation, religious beliefs, and social and environmental trust [4]. In December 2021, the Caribbean area had 2,193,737 confirmed COVID-19 cases, with a case fatality rate (CFR) of 1.34% [5]. However in Ethiopia show as of March 24, 2022, the government had tested almost 4 million suspects, or less than 4% of the total population compared to the above [6], there had been confirmed positive cases, and 1.6% of these had died [7]. All countries are obligated to work together to address the COVID-19 pandemic since it is seen as a global threat. World Health Organization (WHO) and other research teams and healthcare specialists around the world for their extraordinary dedication to vaccine research, development, and production, COVID-19 vaccines were developed in the shortest time in vaccination history [8].

A minimum of 2 billion vaccine doses will be distributed in 2021 by the COVID-19 Vaccines Global Access (COVAX) facility to the afflicted countries worldwide, with at least 1.3 billion doses sponsored by donors for the 92 low-income countries [9]. COVID-19 vaccines are essential for preventing and controlling the disease vaccines are one of the most effective and affordable health interventions for preventing infectious diseases [10]. Under the current global distribution plan, Ethiopia argues that vaccinating 60% of the population by the end of 2022 [11]. A sufficient vaccination rate can slow the spread of the virus within the population. According to estimates, when a vaccine is 100% efficient, 67% of the population must be immunized to attain herd immunity [12].

If a large part of the population becomes infected, the healthcare infrastructure will be severely strained, and over 536 million confirmed cases and over 6.3 million deaths have been reported globally [13], The most prevalent mechanisms of transmission are coughing, sneezing, droplet inhalation, and contact with oral, nasal, and eye mucous membranes resulting in the transmission of viruses to other sites the need for a large number of vaccines is necessary to stop the spread of COVID-19 [14]. Despite the pandemic, there remains a lot of doubt over the effectiveness and safety of vaccines [15]. A crucial step in putting an end to the pandemic is the development of a safe and effective COVID-19 vaccine [16]. It is crucial to make sure that present and future COVID-19 vaccines are widely identified, required, and accepted to safeguard individual health, protect the most vulnerable people, reopen social and economic life, and maybe achieve population health and safety through vaccination. Those who have been overwhelmed by the disease’s loss of life and livelihoods have new hope thanks to the introduction of the COVID-19 vaccine [17, 18].

The COVID-19 vaccines from Pfizer and Moderna both provide about 95% protection [19]. There are currently over 125 vaccine candidates, 365 vaccine studies in progress, and 18 COVID-19 vaccines approved by at least one country around the world [20]. When it comes to preserving the public’s health, the invention of vaccines is regarded as one of the greatest human achievements [21, 22]. Vaccination is the most efficient method of fighting COVID-19, but until now, it has been constrained by people and groups who refuse vaccination [23].

The effectiveness of the COVID-19 vaccine program is ultimately determined by the population’s vaccination uptake. The last ten years have seen a sharp increase in vaccine hesitancy, a complex notion that is described as a refusal, uncertainty, or delay in accepting vaccines notwithstanding vaccine availability [24, 25].

The COVID-19 vaccine misinformation that is widely disseminated on social media may be the primary cause of vaccine-related anxiety [9, 26], concerns about vaccination safety, negative vaccination experiences in the family, and moral or religious objections can all contribute to vaccine anxiety [23]. Due to its incredible rise, vaccination hesitancy is today viewed by the WHO as a major threat to global health [27, 28].

The key factor contributing to vaccination anxiety, particularly about the COVID-19 vaccine, is how quickly it was developed, given a false impression that it had not undergone significant safety and efficacy testing [29], numerous sources claim that vaccine apprehension has been linked to religious beliefs, individual opinions, and safety worries due to widespread misconceptions regarding the connection between vaccines and autism, brain damage, and other disorders in children [30]. According to global health indices, nations with smaller populations, greater government spending on healthcare, and strong public governance have performed the best in fighting the current COVID-19 pandemic [31].

There is a limited study in southwest Ethiopia and no scientific proof of such an attitude towards COVID-19 in this area. This is crucial to determine the level of attitude and prevention measured for the COVID-19 vaccine and its related factors at the Gurage Zone community level in southwest, Ethiopia in 2022.

## Methods and material

### Study design, setting, and period

A cross-sectional study was conducted in the community. This study was conducted in the Gurage Zone, which is one of the administrative regions in southern Ethiopia. Gurage Zone is made up of 13 districts and two local governments. It is 158 kilometers away from the capital city of Addis Ababa. The zone has a total population of 1,279,646, with 657,568 women, according to the 2007 national household census. Eight hospitals service all persons found in Garage Zone (six public and two private). All hospitals provide comprehensive emergency care, including treatment for COVID-19. In addition, the Gurage Zone has 74 health centers that provide basic prevention and medical services. This research was conducted from November 15th to December 15th, 2021.

### Source and study population

All adult residents of Gurage Zone in Southern Ethiopia age 18 years or older were the source population and all those who were randomly chosen were the study population.

### Inclusion and exclusion

The adult population of the Gurage Zone who were living included in the study, however, unable to communicate, and seriously ill were excluded from the study.

### Sample size determination and sampling technique

The sample size was estimated using a single proportion formula from a study conducted at Gondar University, Ethiopia at 31.4% [9] marginal error (d) 0.05, with a 95% confidence interval. By adding a 10% non-response rate, the final sample size becomes 364.


$$n=\frac{\left(Za/2)2\;p(1-p\right)}{d2}$$

$$n=n=\frac{\left(1.96)2.314(1-.314\right)}{(0.05)2}=331$$

### Study variable

#### Dependent variable

Attitude toward vaccine.

#### Independent variables

Socio-demographic factors (age, sex, residence, educational status, occupation, marital status, religion, and estimated monthly income), respondents’ attitude status, media availability, and diagnosed medical health problem (comorbidity).

### Operational definitions

#### Attitude to receive COVID-19 vaccine

Refers to a person’s readiness to receive the COVID-19 vaccine and is measured by six items with a three-point Likert scale. The higher summed score indicates a higher attitude to receive the COVID-19 vaccine.

#### Positive attitude

Respondents were asked "Does the newly discovered COVID-19 vaccine is safe?" and were given a score of 1 if they answered "agree," and 0 if they answered, "undecided and disagree." For each item, the correct answer received a "1," while the erroneous response received a "0" For the other five attitudes-related items, the same style of asking and scoring was used. Respondents who scored above or equal to the mean value of the sum of attitude-related questions were considered to have a positive attitude.

#### Negative attitude

When study participants respond to less than 50% of the above attitude questions.

### Data collection instrument and procedure

A pre-tested, standardized interviewer-administered questionnaire was used to collect the data. After a detailed examination of numerous similar pieces of literature, the questionnaire was developed. It includes socioeconomic data, attitude-related variables, knowledge-related variables, personal health-related, and attitudes to accept the vaccine. The data collectors who have BSc nurses and spoke the local language were used to collect the data under the supervisors of MSc nurses. The data were obtained at the household level through face-to-face interviews and administered questionnaires. The household head or anyone over the age of 18 years old explains all the details of the study before giving them to participate.

### Data processing and analysis

The data were checked for their completeness manually and then entered in Epi data version 3.1 and exported to analyze using SPSS version 23. Bi-variable and multivariable logistic regression analyses were employed in conjunction with descriptive statistics to determine the variables that were related to the community’s level of acceptance of the COVID-19 vaccine. In a multivariable logistic regression analysis, variables that had a bi-variable logistic analysis p-value of less than 0.25 were the candidate. To demonstrate the strength of the association, the crude odds ratio (COR) in bi-variable logistic regression analysis and adjusted odds ratio (AOR) in multivariable logistic regression with the associated 95% confidence interval. In multivariable logistic regression analysis, factors were considered statistically significant when the p-value < 0.05.

### Data quality control

Data quality was ensured through careful design of the data collection tool, appropriate modification, appropriate recruitment, and one-day training for data collectors and supervisors on the purpose of the study, selection of study participants, maintaining the confidentiality of the collected data, filling out the data collection form, and managing and monitoring data quality. Pretest was done on 5% of study subjects and modification was made accordingly. The entire time that the data was being collected, investigators and supervisors provided intensive supervision. To assess the reliability of the questionnaire, Cronbach’s alpha was utilized, and it came back at 0.77, representative of internal consistency.

## Result

### Socio-demographic characteristics

The study included 360 participants, with a response rate of 98.90%. Out of the total number of respondents, 192 (53.3%) were males, and the majority of the participants 314 (87.2%) were married. 136(37.8%) primary school and only 36(10%) secondary and above educational status. The mean age of participants was 52.82 (SD ± 15.2) years. being orthodox 163 (45.3%) 142 (39.4%) Muslim, and 32 (8.9%) protestant. The participants’ average monthly income was 4820 ETB, with 277 (76.9%) living in urban (Table 1).

**Table 1:** Socio-demographic characteristics of study participants in Gurage zone 2021 (n = 360).

Variable	Category	Frequency	Percentage
Sex	Male	192	53.3
	Female	168	46.7
Age in years	18–29	3	0.8
	30–39	49	13.6
	>40	308	85.6
Residence	Urban	277	76.9
	Rural	83	23.1
Religion	Orthodox	163	45.3
	Muslim	142	39.4
	protestant	32	8.9
Ethnicity	Gurage	161	44.7
	Amara	135	37.5
	Oromo	51	14.2
	Other ethnicities	13	3.6
Marital status	Single	46	12.8
	Married	314	87.2
Educational status	Can’t read and write	88	24.4
	Read and write	100	27.8
	Primary school	136	37.8
	Secondary and above	36	10
Family monthly income ETB	<3000	113	31.4
	3001–5000	97	26.9
	5001–10000	104	28.9
	>10001	46	12.8

Others© = (Tigray, Gambella,silte, kaffa).

### Source of information about the COVID-19 vaccine

**Fig 1:**
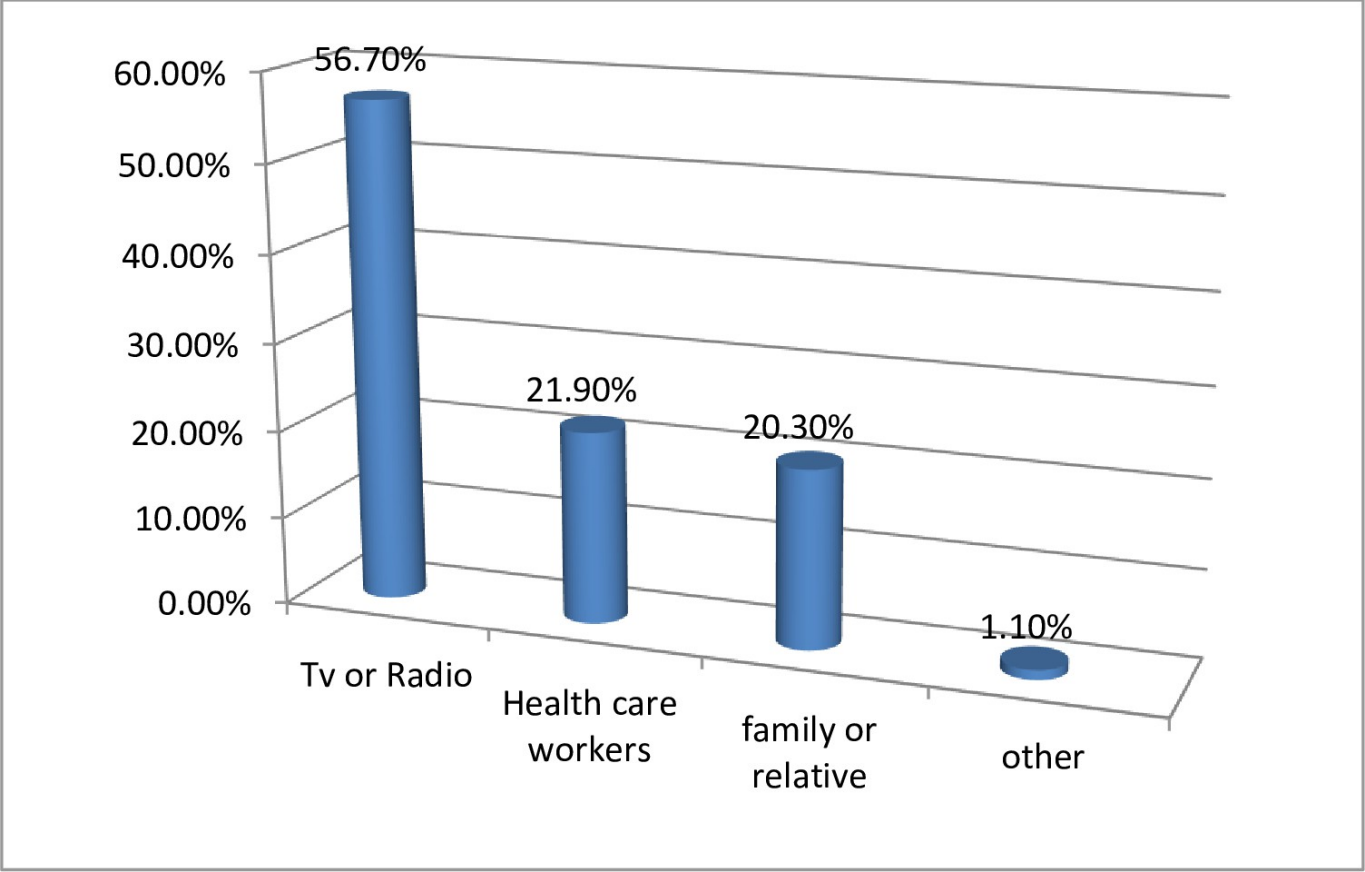
Source of information you get about covid-19 vaccine 2021.

Concerning the source of information on the development of the COVID-19 vaccine, 204 (56.7%) participants heard from the media, 79 (21.9%) participants were from a healthcare worker, 73 (20.3%) participants were from a family or friends, and 4 (1.1%) participants were from other sources (Fig 1).

### Attitude toward COVID-19 vaccination

**Fig 2:**
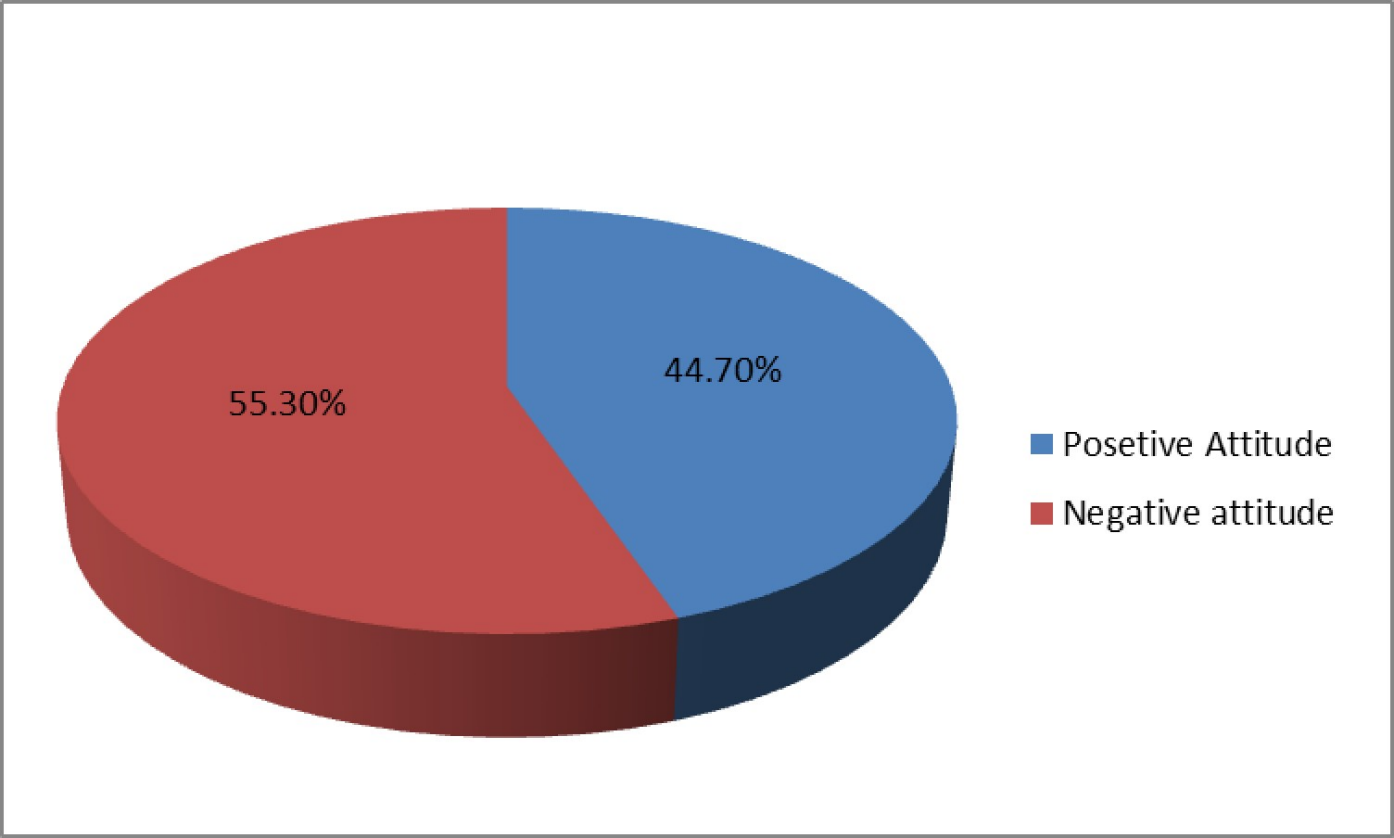
Attitude toward receiving the 2021 COVID-19 vaccine from the Gurage zone.

Out of 360 study participants, 44.7% (95% CI; 39.7–50.3%) of participants have a favorable attitude toward COVID-19 vaccination. Even if a vaccine was available in Ethiopia, more than half of the remaining 199 (55.3%) had a negative attitude against any COVID-19 vaccine (Fig 2). The vaccination is believed to be safe by 112 (31.1%) of participants, whereas the vaccine is not believed to be safe by over half of the participants, 203 (56.4%). Approximately half 170 (47.2%) of participants believe the vaccine itself causes disease, and 92 (25.6%) of participants we don’t know about the vaccine. its related consequence can be reduced by 84 (23.3%) by vaccination. 100 (27.8%) individuals disagree that vaccines reduce problems, and 176 (48.9%) participants are unaware of vaccination effectiveness (Table 2).

**Table 2:** Distribution of respondents’ frequency concerning their attitude toward the COVID-19 vaccine at Gurage Zone 2021.

Variables	Category	Frequency	Percent
New COVID-19 vaccinations are safe.	agree	112	31.1
	disagree	203	56.4
	I’m not sure.	45	12.5
Fear of vaccine side effects.	agree	105	29.2
	disagree	162	45
	I’m not sure.	93	25.8
The vaccine itself causes COVID -19 disease	agree	170	47.2
	disagree	98	27.2
	I’m not sure.	92	25.6
COVID-19 is not that serious a concern in my work or residential area	agree	83	23
	disagree	204	56.7
	I’m not sure.	73	20.3
A family member diagnosed with COVID-19 disease	agree	40	11.1
	disagree	192	53.3
	I’m not sure.	128	35.6
Vaccination decreases getting COVID-19 or its complications	agree	84	23.3
	disagree	100	27.8
	I’m not sure.	176	48.9

### Factors associated with the acceptance of a covid-19 vaccine

Bivariate and multivariate logistic regression analysis was used to examine the factors influencing the uptake of COVID-19 vaccinations. All variables with a p-value of less than 0.25 were included in the multivariable regression model. Age, gender, and marital status, do you believe you would decrease from COVID-19 infection, have a chronic illness, you believe that vaccines will stop pandemics, perceive potential vaccine harm, issue health worker opinion, and are generally against vaccination, given its rapid development, it is unreliable. Lack of information about the vaccine’s safety, the belief that the COVID-19 vaccine should be given to everyone, the practice of other preventative measures, encouragement of others to get the vaccine, fear of contracting the COVID-19 disease, and a lack of information were included in the multivariable regression analysis.

All significant factors in binary logistic regression were a candidate in multivariable logistic regression analysis. Perceived potential vaccine harm [AOR: 1.85; 95% CI (1.15–2.96) P 0.011] have a more optimistic attitude than their peers and have a chronic illness [AOR: 3.22; 95% CI (2.02–5.14) P 0.000] Community believes vaccine will stop pandemic [AOR: 2.02; 95% CI (1.27–3.22) P 0.003)] compared to their counterparts. Average monthly income 3001–5000 [AOR: 0.54; 95% CI (0.30–0.97) P 0.03)], Average monthly income 5001–10,000 [AOR: 0.48; 95% CI (0.27–0.86) P 0.01)] were statistically significantly associated with a positive attitude towards COVID-19 vaccination (Table 3).

**Table 3:** Participants with factors associated with acceptance of COVID-19 vaccine in the Gurage zone, Southern Ethiopia, 2021.

Variable	Category	COR (95%CI)	AOR (95%CI)	P value
Marital status	Married	1.72(.93–3.22)	1.9(.97–3.72)	0.06
	single	1	1	
Do you think you are likely to die if infected by COVID-19	Yes	.69(.41–1.16)	.59(.34–1.05)	0.07
	No	1	1	
Perceived potential vaccine harm	Yes	1.68(1.10–2.57)	1.85(1.15–2.96)	0.011
	No	1	1	
Have you a chronic disease	Yes	3.00(1.94–4.66)	3.22(2.02–5.14)	0.000
	No	1	1	
Community believe vaccine stop pandemic	Yes	.53(0.35–0.82)	2.02(1.27–3.22)	0.003
	No	1	1	
Income category	<3000	1	1	
	3001–5000	1.89(.94–3.80)	0.54(0.30–0.97)	0.03
	5001–10000	1.19(.58–2.43)	0.48(0.27–0.86)	0.01
	>10001	.93(.46–1.90)	.52(0.25–1.11)	0.09

Significance at P value < 0.25.

Significance at P value < 0.05.

AOD: Adjusted Odd Ratio, COR: Crude Odd Ratio CI: Confidence Interval.

## Discussion

Many COVID-19 vaccine candidates were created, and positive results from abundant clinical investigations led to the licensure of numerous vaccines used in various nations [32]. Vaccines against COVID-19 have been created as "the ideal solution" to terminate the current pandemic. Following several encouraging clinical trials, many countries have approved several vaccines for use in Ethiopia’s vaccine programs [33].

The overall prevalence of positive attitudes toward COVID-19 was found 161 (44.7%) with a 95% CI (39.7–50.3%). The results of this study were in line with the study done at Wolaita at 45.5% (30), Addis Ababa at 48.2% (31), Debre Tabor at 42.3% (32), and Nigeria at 50.2% (33). However, this finding was higher than the study conducted in Gondar 32.2% (2), Pakistan 37.80% (34), and Jordan 37.4% (35). The possible difference could be found to differ in socio-demographic characteristics, religious practices and beliefs, and uncertainties during the relatively short period of new vaccine development.

This study finding was less than the study conducted in Bangladesh 74.5% [34], Ghana 51% [35], the United Kingdom 63.5% [36], Gondar 54.8% [26], Canada 79.8% [37], India 64.5% [38], and Israel 79% [39]. This disparity could be attributed to differences in economic factors, educational attainment, and access to information among the study populations, the methodology and study settings, and the availability and accessibility of health services infrastructure may all contribute to the inability to provide for the community in south Ethiopia.

As an illustration, the current study was carried out at the community level, where COVID-19 modifying measures were implemented, such as the use of face masks, maintaining a safe physical distance, and hand washing. According to this poll, the majority of patients believe that COVID-19 vaccinations are a community that trusts in the efficacy of vaccines to stop a pandemic and that health officials won’t be able to vaccinate the majority of Ethiopians.

Three-quarters of the study participants claimed that the community does not believe in the safety of vaccines to prevent pandemics and that health officials will not be able to vaccinate the majority of the population. More than half of the study participants had negative attitudes toward COVID-19 vaccines [40]. The WHO states that vigorous determinants of trust in the health system include people’s attitudes toward the competence of health authorities, the objectivity of the information and actions provided, the consistency of their messages and actions, and their sincerity by demonstrating transparency and empathy through actions. One of the most important ways to improve people’s attitudes toward vaccines is to involve policymakers in overcoming suspicion and fostering public confidence [41].

The current study found that people who perceived potential vaccine harm [AOR: 1.85; 95% CI (1.15–2.96) P 0.011] have a more optimistic attitude than their counterparts. This study is in line with those previously conducted at Kuiate [42], and the United Kingdom [43]. This resemblance could be due to many factors, including suspicion about vaccine-related complications. Participants who believe the vaccine will stop the epidemic had a higher [AOR: 2.02; 95% CI (1.27–3.22) P 0.003] than their counterparts. This study is similar to one conducted in Beijing China [44]. Participants with chronic disease (86.4%) had a positive attitude toward accepting the vaccine [AOR: 3.22; 95% CI (2.02–5.14) P 0.000], this study is similar to those previously conducted in Australia [45], Hong Kong [46]. This similarity could be due to the majority of study participants. were aware that patients with underlying chronic medical conditions were at a higher risk of morbidity by COVID-19 than their counterparts.

The income status of the respondents became one of the factors which affect the acceptance of the COVID-19 vaccine. This study is consistent with the findings in the USA, England, and Japan [47–49]. The possible reason could be vaccine hesitancy by occupation, with non-clinical personnel having the highest likelihood of vaccine hesitancy, a unique finding of this study that may be related to wealth. Financial incentives, such as cash rewards after vaccination and tokens to assist with transportation costs to vaccination sites, are highly suggested.

## Conclusion and recommendation

According to the outcomes of this study, the acceptability of a COVID-19 vaccination was low. Acceptance of the COVID-19 vaccinations was found to be substantially linked to Potential vaccine risk, chronic disease, community belief that vaccination will stop the pandemic, and income should be considered toward the attitude toward a vaccine. The COVID-19 vaccination attributes can be used to influence localized interventions and strategies to improve the community, attitude, and attitude toward vaccination. To improve community acceptability of the COVID-19 vaccination, the government should collaborate with other stakeholders to implement community awareness about the vaccine’s benefits disseminated through different media.

### Strength and limitation

The study’s strength is in identifying the determinants of attitudes toward the COVID-19 vaccine, which can provide useful information to policymakers and other stakeholders. However, the study has the disadvantages of a cross-sectional study design in that establishing a causal relationship between the parameters investigated and vaccination acceptance may be challenging.

## List of abbreviations

AOR: Adjusted Odds Ratio
CDC: Centers for Disease Control and Prevention
COR: Crude Odds Ratio
COVID-19: Corona Virus Disease-2019
SNNP: South Nation Nationalities, and Peoples
SPSS: Statistical Package for Social Science
WHO: World Health Organization
